# Examining the roles of travel distance, medical mistrust, and cancer fatalism in the uptake of clinical cancer prevention among women in rural and urban US communities: A secondary data analysis

**DOI:** 10.1016/j.pmedr.2024.102611

**Published:** 2024-01-13

**Authors:** Jane-Frances Aruma, Madison Hearn, Veronica Bernacchi, Jennifer L. Moss

**Affiliations:** a Penn State College of Medicine, The Pennsylvania State University, State College, PA, USA; b Penn State College of Medicine, The Pennsylvania State University, Hershey, PA, USA

**Keywords:** Rural health, Urban health, Disparities, Cancer prevention, Cancer screening, Primary care, Cancer disparities

## Abstract

**Introduction:**

Rural adults are less likely to receive cancer screening than urban adults, likely due to systematic differences in community- and individual-level factors. The purpose of this study was to analyze the relative contributions of rurality, travel time, medical mistrust, and cancer fatalism in explaining uptake of clinical cancer prevention services.

**Methods:**

We conducted a secondary data analysis of 2019–2020 survey data from women, ages 45–65, in rural and urban counties in central Pennsylvania, examining rurality, travel time to a primary care provider, medical mistrust, and cancer fatalism, as well as uptake of guideline-recommended colorectal cancer screening, cervical cancer screening, and preventive check-up. Final models used multivariable logistic regression to assess the relationships among study variables, controlling for participant demographics.

**Results:**

Among 474 participants, 48.9 % resided in rural counties. Most participants had received clinical cancer prevention services (colorectal cancer screening: 55.4 %; cervical cancer screening: 82.8 %; preventive check-up in the last year: 75.4 %). Uptake of services was less common among participants with higher medical mistrust (colorectal cancer screening: adjusted odds ratio [aOR] = 0.87, 95 % confidence interval [CI] = 0.76–1.00; cervical cancer screening: aOR = 0.79, 95 % CI = 0.63–1.00; last-year check-up: aOR = 0.74, 95 % CI = 0.63–0.88).

**Conclusions:**

Patient attitudes, particularly medical mistrust, may contribute to rural/urban disparities in clinical cancer prevention among women. Community- and individual-level interventions are needed to improve cancer outcomes in rural areas.

## Highlights

• Rural residents are less likely than urban to receive clinical cancer prevention.
• We compared several competing explanations for this difference.
• Clinical cancer prevention was negatively associated with medical mistrust.
• This association remained when controlling for travel distance, fatalism, rurality.

## 1 Introduction

Despite advancements in cancer screening and treatment, cancer remains the second leading cause of death in the United States (1). Early diagnosis and treatment can improve cancer patients’ outcomes, such as quality of life and disease progression, but disparities exist in the uptake of clinical cancer prevention services (Meilleur et al., 2013). For example, the uptake of clinical cancer prevention services is suboptimal in rural communities. Rural residents have lower uptake of clinical cancer prevention services including tests for breast cancer (Doescher and Jackson, 2009, Larson and Correa-de-Araujo, 2006, Casey et al., 2011), cervical cancer (Larson and Correa-de-Araujo, 2006, Casey et al., 2011), and colorectal cancer (Casey et al., 2011). This may be due to factors that are common among rural residents, such as inadequate health insurance, lower income levels, longer distance to screening facilities, poor literacy, and limited knowledge of risks and benefits of cancer screening (Charlton et al., 2015). Low rates of uptake of clinical cancer prevention services in rural areas may contribute to the elevated incidence and mortality rates of cancer in rural compared to urban communities (Unger et al., 2018).

Underutilization of clinical cancer prevention among rural patients persists even after controlling for demographics and healthcare market characteristics (Larson and Correa-de-Araujo, 2006, Casey et al., 2011), suggesting that other factors may explain rural/urban disparities in uptake. Community- and individual-level characteristics, such as travel distance (Larson and Fleishman, 2003, Probst et al., 2007, Syed et al., 2013, Baron et al., 2008), medical mistrust (Adams et al., 2017), and cancer fatalism (Keller et al., 2021), vary by rurality and may undermine motivation and participation in cancer prevention. In terms of travel distance, the low availability of healthcare resources in rural communities necessitates longer travel distance/time for rural residents than urban residents (Larson and Fleishman, 2003, Syed et al., 2013), which serves as a major barrier for accessing healthcare services (Larson and Correa-de-Araujo, 2006).

Medical mistrust is a lack of trust in the motives of healthcare providers and organizations (Omodei and McLennan, 2000). Medical mistrust is predictive of health service underutilization (LaVeist et al., 2009), delayed seeking of medical care (LaVeist et al., 2009), and decreased use of preventive services (Williamson and Bigman, 2018). Research analyzing medical mistrust has focused mainly on racial/ethnic differences (Williamson and Bigman, 2018, Schwei et al., 2014), but a few studies have characterized medical mistrust in rural populations (McAlearney et al., 2012, Nelms et al., 2014, Statz and Evers, 2020). It is possible that medical mistrust in rural communities stems from concerns about discrimination and lack of confidentiality (Douthit et al., 2015).

Cancer fatalism, or the belief that developing (and dying from) cancer is an uncontrollable event, contributes to feelings such as pessimism and powerlessness, as well as avoidance of information-seeking, cancer screening, and healthy behaviors (Keller et al., 2021, Niederdeppe and Levy, 2007). Higher cancer fatalism has been reported among people with lower education levels (Keller et al., 2021, Niederdeppe and Levy, 2007) and lower health literacy (Powe and Finnie, 2003), who are more likely to be residents of rural communities. In particular, rural and Appalachian communities are characterized by high levels of cancer fatalism (Jensen et al., 2022, Vanderpool et al., 2019, Vanderpool et al., 2015). Frequent cycles of cancer diagnoses and consequent deaths within one’s community may serve to strengthen cancer fatalism in rural communities (Keller et al., 2021, Powe and Finnie, 2003). These beliefs likely obscure the benefits of clinical cancer prevention services (Espinosa de Los and Linda, 2011).

The aim of this study is to further outline factors (i.e., travel time, medical mistrust, and cancer fatalism) contributing to disparities in utilization of clinical cancer prevention services among women living in rural versus urban communities, controlling for pertinent individual-level characteristics. Understanding the roles of these factors is particularly important for informing future strategies to improve uptake of clinical cancer prevention in these underserved populations.

## 2 Methods

### 2.1 Sample and survey design

This study is a secondary analysis of a survey focused on understanding cancer screening behaviors. The survey was administered from November 2019 - May 2020 to residents of the 28-county catchment area of the Penn State Cancer Institute. Inclusion criteria for participants included being 1) female, 2) age 45–65 years old, 3) English-speaking, and 4) residing in the catchment area. It should be noted that the majority of recruitment and data collection took place during the early months of the COVID-19 pandemic, which could have impacted responses to survey items. Importantly, though, the primary study outcomes (described below) were retrospective, focusing on receipt of clinical cancer prevention services for up to 10 years before study participation; some of these behaviors therefore would have been minimally impacted by the pandemic. Participants were recruited through social media outreach, primarily via Facebook and Instagram ads that linked to the study page hosted on the Penn State Cancer Institute website. For full details on study design, see the primary manuscript (Centers for Disease Control and Prevention, 2021). Overall, 474 participants enrolled in this study. Each participant provided verbal consent before completing the survey over the phone or online. Participants received a $15 gift card upon survey completion. Data collection and analysis for this project were approved by the Pennsylvania State University Human Research Proection Program.

### 2.2 Measures

*Demographic characteristics* included participants’ self-reported race/ethnicity, educational attainment, annual household income, marital status, and insurance status. We also assessed pertinent healthcare variables such as personal cancer history and hysterectomy. *Rurality* was determined based on county of residence, which we classified as urban or rural according to the USDA rural–urban continuum codes (U.S. Department of Agriculture, 2023), such that counties with codes of 1–3 (“metropolitan”) were classified as urban, and counties with codes of 4–9 (“non-metropolitan”) were classified as rural.

*Correlates of cancer prevention* included travel time to the primary care provider’s (PCP’s) office, medical mistrust, and cancer fatalism. Travel time was assessed with the survey item, “On a typical day, how long, in minutes, would it take you to drive from your home to your primary care provider’s office?” (Participants who did not have a primary care provider skipped this question.) Medical mistrust was assessed with three items (Turgeon et al., n.d.) (Cronbach’s alpha = 0.41, which is similar to internal consistency achieved in other studies using this scale (Moss et al., 2022)); responses for the three items were summed to create a medical mistrust score ranging from 0 to 9, with higher scores indicating greater mistrust. Cancer fatalism was assessed with a 3-item scale developed for the NCI Health Information National Trends Survey (National Cancer Institute, 2023) (Cronbach’s alpha = 0.62, which is similar to internal consistency achieved in other studies using this scale (33)); responses for the three items were summed to create a cancer fatalism score ranging from 0 to 9, with higher scores indicating greater fatalism.

*Clinical cancer prevention services* were self-reported timely receipt of colorectal cancer screening, cervical cancer screening, and preventive check-up. Participants were classified as up-to-date with screening if they received a screening test within the recommended screening interval, according to the American Cancer Society guidelines (American Cancer Society, 2024) for colorectal cancer screening (e.g., colonoscopy within the last 10 years) and for cervical cancer screening (e.g., Pap test within the last 3 years; excluding participants who had had a hysterectomy). Finally, participants reported the last time they had attended a preventive healthcare visit (“check-up”), which we classified as in the last year or not.

### 2.3 Statistical analysis

First, we generated descriptive statistics of demographic characteristics of the study sample. We used chi-square tests to assess bivariate differences in demographics by rurality. Then we calculated the (1) mean and standard error for each correlate of cancer prevention and (2) frequency of uptake for each clinical cancer prevention service. We used *t*-tests and chi-square tests, respectively, to assess bivariate differences in correlates of cancer prevention and uptake of services by rurality.

We analyzed the unadjusted association between rurality and each outcome using bivariate logistic regression. Then, we constructed multivariable models to estimate the adjusted associations between rurality, correlates of cancer prevention, and participant demographics with each outcome. Analyses were conducted in SAS v9.4 and used a two-sided *p*-value of 0.05.

## 3 Results

The 474 participants were split across urban (50.2 %) and rural (49.8 %) counties (Table 1). Urban participants were more likely than rural participants to report a race/ethnicity besides non-Hispanic White (urban: 4.6 %; rural: 0.9 %, *p* =.01). Urban participants were also more likely than rural participants to report a lower household income (30.0 % vs. 21.2 %, respectively, *p* =.03), to be non-married (34.1 % vs. 19.6 %, *p* <.001), and to have non-private health insurance (34.0 % vs. 20.8 %, *p* <.01). There was no difference by rurality for participants’ educational attainment, having had a hysterectomy, or personal cancer history.

**Table 1:** Descriptive statistics for study participants (*n* = 474) in urban and rural communities (2019–2020).

	Urban (*n* = 238)		Rural (*n* = 236)		*p*
	*n*	%	*n*	%	
Race/ethnicity					**0.01**
Non-Hispanic white	227	95.4	234	99.2	
Other	11	4.6	2	0.9	
Educational attainment					0.11
High school degree or less	38	16.4	25	11.2	
More than high school degree	194	83.6	199	88.8	
Annual household income					**0.03**
<$50,000	69	30.0	47	21.2	
$50,000 or more	161	70.0	175	78.8	
Marital status					**<0.001**
Not married/living with a partner	79	34.1	44	19.6	
Married/living with a partner	153	66.0	181	80.4	
Insurance status					**<0.01**
Non-private	81	34.0	49	20.8	
Private	157	66.0	187	79.2	
Had a hysterectomy					0.23
No	179	75.2	166	70.3	
Yes	59	24.8	70	29.7	
Personal cancer history					0.59
No	198	83.2	191	81.3	
Yes	40	16.8	44	18.7	

*Note.* Boldface indicates statistical significance (*p* <.05).

On average, participants reported a moderate travel time to their PCP’s office (mean = 17.15, SE = 0.62, range: 1–120), which was shorter for urban participants (mean = 15.73, SE = 0.81) than rural participants (mean = 18.57, SE = 0.92) (*p* =.02) (Table 2). Average medical mistrust scores were 3.06 (SE = 0.07), and they did not differ by rurality (*p* =.51). Finally, average cancer fatalism scores were 4.11 (SE = 0.09), and they did not differ by rurality (*p* =.55).

**Table 2:** Overall and urban/rural differences in correlates of cancer prevention and clinical cancer prevention services among women (ages 45–65 years) in urban and rural communities (2019–2020).

	Overall		By county type				
			Urban		Rural		
***Correlates of cancer prevention***	mean	*SE*	mean	*SE*	mean	*SE*	*p*
Travel time to PCP office, minutes [range: 1–120]	17.15	0.62	15.73	0.81	18.57	0.92	**0.02**
Medical mistrust [range: 0–9]	3.06	0.07	3.02	0.10	3.11	0.10	0.51
Cancer fatalism [range: 0–9]	4.11	0.09	4.06	0.13	4.17	0.11	0.55

***Clinical cancer prevention services***	*n*	%	*n*	%	*n*	%	*p*
Up-to-date with colorectal cancer screening	262	55.4	124	52.3	138	58.5	0.18
Up-to-date with cervical cancer screening^1^	284	82.8	148	83.2	136	82.4	0.86
Last-year check-up	356	75.4	182	76.8	174	74.0	0.49

*Note*. Boldface indicates statistical significance (*p* <.05). *SE* = standard error; PCP = primary care provider.

1 Excludes participants with a hysterectomy (*n* = 129).

### 3.1 Up-to-date with colorectal cancer screening

**Fig. 1:**
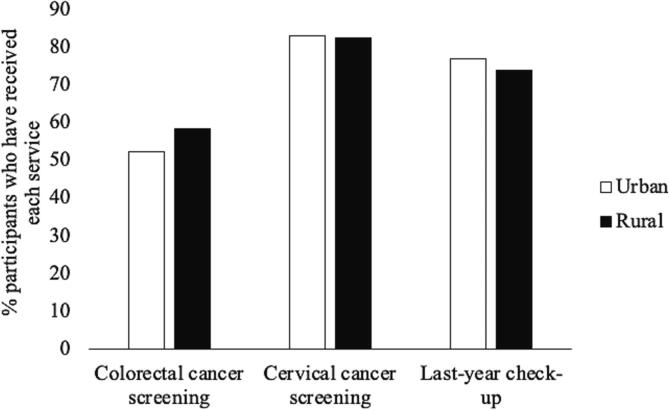
Differences in uptake of clinical cancer prevention services among women (ages 45–65 years) in urban compared to rural communities (2019–202).

Overall, 55.4 % of participants were up-to-date with colorectal cancer screening, which did not differ by rurality (*p* =.18) (Fig. 1). In the multivariable model, controlling for participant demographics, colorectal cancer screening was less common among participants with greater medical mistrust (adjusted odds ratio [aOR] = 0.87, 95 % confidence interval [CI] = 0.76–1.00) (Table 3).

**Table 3:** Bivariate and multivariable relationships between rurality, correlates of cancer prevention, and uptake of clinical cancer prevention services among women (ages 45–65 years) in urban and rural communities (2019–2020).

	Up-to-date with colorectal cancer screening				Up-to-date with cervical cancer screening^1^				Last-year check-up			
	Bivariate		Multivariable		Bivariate		Multivariable		Bivariate		Multivariable	
	OR	95 % CI	aOR	95 % CI	OR	95 % CI	aOR	95 % CI	OR	95 % CI	aOR	95 % CI
Rurality												
Urban	(ref)		(ref)		(ref)		(ref)		(ref)		(ref)	
Rural	1.28	(0.89–1.85)	1.30	(0.87–1.93)	0.95	(0.54–1.67)	0.68	(0.35–1.31)	0.86	(0.57–1.31)	0.76	(0.47–1.20)
Travel time to PCP office (by 10 min)	0.94	(0.82–1.07)	0.92	(0.79–1.06)	1.04	(0.83–1.32)	1.01	(0.79–1.29)	1.00	(0.85–1.17)	1.02	(0.86–1.21)
Medical mistrust	0.85	(0.75–0.96)	0.87	(0.76–1.00)	0.72	(0.59–0.87)	0.79	(0.63–1.00)	0.73	(0.63–0.85)	0.74	(0.63–0.88)
Cancer fatalism	0.97	(0.88–1.08)	0.97	(0.87–1.08)	1.18	(1.01–1.39)	1.28	(1.06–1.55)	1.01	(0.90–1.13)	1.05	(0.92–1.19)

*Note*. Boldface indicates statistical significance (*p* <.05). OR = odds ratio; CI = confidence interval; aOR = adjusted odds ratio; ref = reference category; PCP = primary care provider.

1 Excludes participants with a hysterectomy (*n* = 129).

### 3.2 Up-to-date with cervical cancer screening

Overall, 82.8 % of participants were up-to-date with cervical cancer screening, which did not differ by rurality (*p* =.86) (Fig. 1). In the multivariable model, cervical cancer screening was less common among participants with greater medical mistrust (OR = 0.79, 95 % CI = 0.63–1.00), and screening was more common among participants with greater cancer fatalism (OR = 1.28, 95 % CI = 1.06–1.55) (Table 3).

### 3.3 Last-year check-up

Overall, 75.4 % of participants had a check-up in the last year, which did not differ by rurality (*p* =.49) (Fig. 1). In the multivariable model, last-year check-up was less common among participants with greater medical mistrust (OR = 0.74, 95 % CI = 0.63–0.88) (Table 3).

## 4 Discussion

In this secondary analysis of a dataset focused on uptake of clinical cancer preventive services among women in central Pennsylvania, we explored the relative contributions of three constructs related to rural/urban differences in clinical cancer prevention: travel time to the PCP’s office, medical mistrust, and cancer fatalism. Overall, we found no evidence of rural/urban differences in timely receipt of colorectal cancer screening, cervical cancer screening, or preventive check-up. This finding is notably inconsistent with the extant literature, which has highlighted rural–urban disparities in cancer incidence, screening, diagnosis, and care. (Doescher and Jackson, 2009, Larson and Correa-de-Araujo, 2006, Casey et al., 2011, Zahnd et al., 2018, Liff et al., 1991) Further, controlling for the other correlates of clinical cancer prevention (i.e., travel time, medical mistrust, and cancer fatalism) did not meaningfully impact the relationships between rurality and cancer prevention. One possible explanation is that check-ups, cervical cancer screening, and certain tests for colorectal cancer screening can be performed in most primary care settings, which are more accessible in rural communities than are specialized facilities (Moss et al., 2019), which could minimize the impact of healthcare access on receipt of these services.

Across outcomes, higher levels of medical mistrust were associated with lower uptake of clinical cancer prevention services. This pattern persisted even after controlling for rurality, travel time, cancer fatalism, and participant demographics. La Veist et al. showed that patient mistrust toward the health care system can lead to failure to heed medical recommendations and attend subsequent medical appointments (LaVeist et al., 2009), which could explain the negative relationship between medical mistrust and uptake of preventive services. Much of the existing research on medical mistrust focuses on differences by race/ethnicity (Adams, et al., 2017, LaVeist et al., 2009, Williamson and Bigman, 2018, Schwei et al., 2014, Jaffee et al., 2021, Boyas and Valera, 2011), but some studies have also shown that rural patients may have more medical mistrust (Spleen et al., 2014, Caston et al., 2022, López-Cevallos et al., 2014, Hall et al., 2018), which can serve as a barrier to cancer screening (Hausmann et al., 2011). Medical mistrust may emerge from patients’ experiences of healthcare discrimination (Shelton et al., 2017), which patients often attribute to disease status and income level (Caston et al., 2022). In our study, medical mistrust was generally low and about the same for rural vs. urban participants. Even so, medical mistrust was the most important correlate we found for clinical cancer prevention. Additional research is needed to engage with patients to decrease medical mistrust and increase uptake of clinical cancer prevention services. This could be done by improving patient-provider communication, utilizing existing social support networks (Hausmann et al., 2011), employing lay health advisors (Segel and Lengerich, 2020), and working to mitigate personal and systemic discrimination.

In our sample, rural participants reported longer travel times to their PCP’s office than urban participants. This finding is consistent with other studies reporting rural/urban differences in travel time (i.e., minutes travelled) (Charlton et al., 2016, Ambroggi et al., 2015), as well as studies demonstrating rural/urban differences in travel distance (i.e., miles travelled) (Amiri et al., 2023, Sahar et al., 2022, Adams et al., 2020, Fuzzell et al., 2021). Generally, previous studies have demonstrated that increased travel time and distance serve as barriers to uptake of clinical services related to cancer. However, in our study, increased travel time was not associated with uptake of clinical cancer prevention services, after controlling for other individual-level barriers to care. This could potentially be as a result of the increased usage of mobile screening units in the surrounding community, which have demonstrated effectiveness at increasing cancer screening in rural communities (Kobayashi and Smith, 2016), as well as population-level reductions in healthcare-seeking behaviors during the early part of the COVID-19 pandemic that coincided with our study, which could have minimized the impact of travel time on access to care. Increasing healthcare accessibility to members of rural communities by decreasing travel time is an important part of a multilevel approach to improving rural cancer prevention and control.

Fatalistic beliefs about cancer evoke unfavorable responses towards cancer information seeking (Clarke et al., 2021), cancer vaccination (Vanderpool et al., 2015), and cancer screening services (Marván et al., 2016). Among our participants, cancer fatalism did not differ between urban and rural participants. Although the study conducted by Moss et al. (2019) showed similar findings, this is in contrast to other studies that have demonstrated that fatalistic beliefs are more prominent in rural populations (Jensen et al., 2022). Also contrary to our expectations based on other studies (Marván et al., 2016), our findings demonstrated a positive relationship between cancer fatalism and being up-to-date with cervical cancer screening. The same pattern was not observed with being up to date with colorectal cancer screening or with having had a checkup in the last year. Additional research is needed on fatalism (and related concepts, e.g., cancer fear) and how it relates to different cancer screening behaviors.

Study strengths include expanding upon previous literature by further exploring the relative, combined contributions of travel distance, medical mistrust, and cancer fatalism to rural/urban disparities in uptake of clinical cancer prevention services. Study limitations include a sample that is demographically homogenous, which limits the generalizability of our study findings, and the self-reported nature of the outcome variables, which could be subject to recall bias. In addition, this study leveraged a convenience sample of participants who self-selected into the study; these participants may vary systematically from the general population in several ways (e.g., greater interest in health, higher e-literacy), which could have biased our results. Future studies should address these limitations, and conduct multilevel analysis to examine environmental, social, and contextual factors that may overlap with patient attitudes and healthcare access to contribute to rural/urban disparities in cancer screening.

## 5 Conclusion

We did not find evidence of rural/urban disparities in clinical cancer prevention among a sample of female patients in central Pennsylvania. Other factors, primarily medical mistrust, are more strongly associated with being up-to-date with colorectal cancer screening, cervical cancer screening, and having a recent preventive healthcare check-up visit. Medical mistrust may prevent rural and urban patients from accessing clinical services. Community and individual-level interventions are needed to address medical mistrust and to improve access and uptake of clinical cancer prevention services. In the long-term, these activities may improve reduce cancer incidence and mortality for residents of both rural and urban communities.

## Funding

This study was supported by an Institutional Research Grant, IRG-17-175-04 (PI: Moss), from the American Cancer Society.

## CRediT authorship contribution statement

**Jane-Frances Aruma:** Writing – review & editing, Writing – original draft, Visualization, Investigation. **Madison Hearn:** Writing – review & editing, Writing – original draft, Investigation, Conceptualization. **Veronica Bernacchi:** Writing – review & editing, Writing – original draft, Supervision, Investigation. **Jennifer L. Moss:** Writing – review & editing, Writing – original draft, Validation, Supervision, Software, Resources, Project administration, Methodology, Investigation, Funding acquisition, Formal analysis, Data curation, Conceptualization.

## Declaration of competing interest

The authors declare that they have no known competing financial interests or personal relationships that could have appeared to influence the work reported in this paper.

## Data Availability

Data will be made available on request.
